# Green ultrasound-assisted extraction, purification, characterization and stability kinetics of anthocyanins from *Rosa chinensis Jacq.*

**DOI:** 10.1016/j.fochx.2026.104358

**Published:** 2026-08-24

**Authors:** Naeem Arshad, Aroona Maryam, Zetian Cai, Haowen Meng, Bingxue Wang, Xiaoxue Kong, Haibo Luo, Lijuan Yu

**Affiliations:** a Agro-products Processing Research Institute, Yunnan Academy of Agricultural Sciences, Kunming 650221, China; b School of Food Science and Pharmaceutical Engineering, Nanjing Normal University, Nanjing 210023, China

**Keywords:** Natural pigments, Cyanidin-3,5-O-diglucoside, LC-ESI-MS/MS, Degradation kinetics, Antioxidant capacity

## Abstract

*Rosa chinensis* Jacq. petals are a rich, underutilized source of anthocyanin pigments, but these compounds are highly sensitive to light, temperature, and pH, and conventional extraction methods are often solvent-intensive, energy-demanding, and damaging to pigment stability. A food-grade, green ultrasound-assisted extraction (UAE) strategy was therefore developed and optimized using single-factor experiments followed by a three-factor, three-level Box-Behnken design, to establish an efficient and scalable protocol for recovering rose anthocyanins. Extraction temperature and time were identified as the most influential factors, and the optimal conditions were 60% (*v*/v) ethanol and 40% (*v*/v) aqueous citric acid (2% *w*/*v*), 36.3 °C, a solid-to-liquid ratio of 1:49.4 g mL^−1^, and 23.9 min, yielded 34.99 g kg^−1^ dry weight (DW) anthocyanins. Macroporous resin purification (Amberlite XAD-7HP) enriched the pigment fraction, and LC-ESI-MS/MS confirmed three anthocyanins at MSI confidence Level 2, with cyanidin-3,5-O-diglucoside as the dominant compound. The purified extract exhibited first-order thermal degradation kinetics (E_a_ = 67.50 kJ mol^−1^) and excellent stability under pasteurization conditions (70 °C, t_1/2_ = 12.6 h), along with substantial pigment retention after ten days of light (69.8%) and dark (82.3%) storage. Strong antioxidant activity was confirmed by DPPH and ABTS assays (TEAC of 2036.39 and 1455.15 mmol TE g^−1^ DW, respectively). These findings establish *Rosa chinensis* Jacq. as a stable, potent source of natural anthocyanin colorants and antioxidants with promising potential as functional ingredients in food and nutraceutical applications.

## Highlights

• UAE with acidified ethanol optimized via Box-Behnken RSM yielding 34.99 g kg^−1^ DW.
• Amberlite XAD-7HP purification achieved 79.9% recovery and 4.1-fold enrichment.
• LC-ESI-MS/MS confirmed three anthocyanins at MSI Level 2 in *Rosa chinensis* Jacq.
• Thermal kinetics showed t₁/₂ = 12.8 h at 70 °C and Ea = 67.50 kJ mol^−1^.
• Dark storage retained 82.3% pigment vs. 69.8% under light after 10 d.

## Graphical abstract

Graphical abstract of the anthocyanin extraction optimization process from edible rose petal**.**

**Figure:**
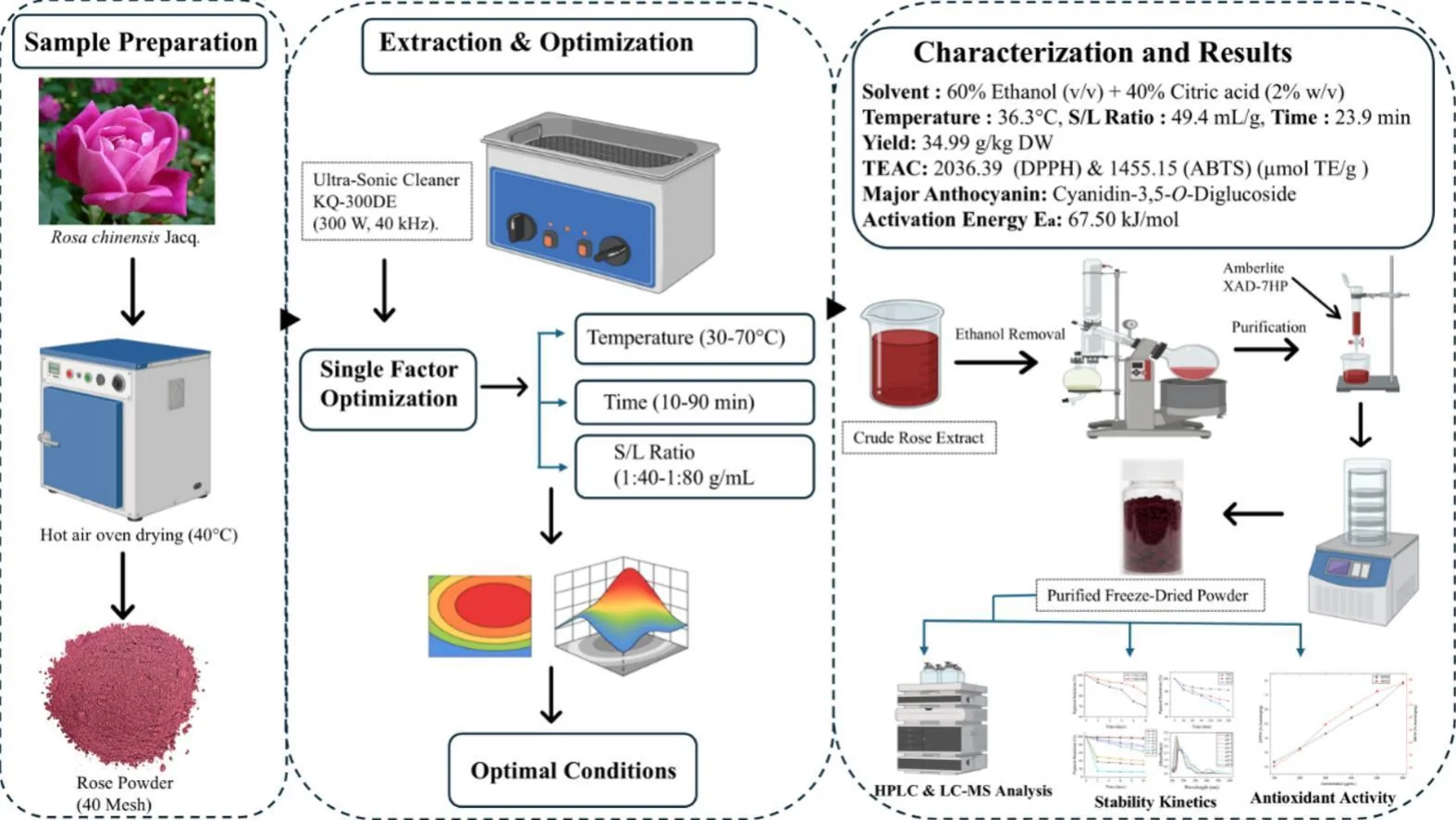

## 1 Introduction

Roses are a rich source of bioactive compounds, particularly anthocyanins. Anthocyanins are a class of water-soluble flavonoids which are responsible for the vibrant red, blue, and purple hues in many fruits, vegetables, and flowers (Benvenutti et al., 2022; Kim et al., 2023). They have gained significant attention in the food and pharmaceutical industries as natural pigments. In addition, researchers have reported that they are used for their potent antioxidant activities and associated health benefits, like anti-inflammatory, anti-diabetics and anti-carcinogenic effects (Jang et al., 2019; Kim et al., 2020). This has increased the demand for efficient and sustainable methods to extract these bioactive compounds from plant sources.

Many conventional methods for anthocyanin extraction, notably mechanical agitation, have been used due to their operational simplicity. However, these methods are often characterized by prolonged extraction times, high solvent consumption, and elevated temperatures, which can accelerate the thermal degradation of anthocyanins, ultimately compromising yield, quality and increased costs (Nunes Mattos et al., 2022). Ultrasound-assisted extraction (UAE) has emerged as a prominent green alternative. The mechanism of UAE is primarily based on acoustic cavitation, which significantly enhances cell wall disruption, improves solvent penetration, and facilitates mass transfer of intracellular compounds into the solvent (Franco-Londoño et al., 2025; Lucas-González et al., 2025).

Methanol and acetone often achieve high yields in analytical settings, but their toxicity limits their application in food products (Chongwilaikasem et al., 2024; Kuppusamy et al., 2023). In contrast, researchers found acidified aqueous ethanol as a safer solvent system that effectively balances extraction efficiency with no toxicity. The ethanol component acts as a permeabilizing agent, disrupting plant cell membranes, while the acidic environment stabilize the anthocyanin molecule in the soluble flavylium cation form, and it helps to break down the plant matrix, thereby enhancing the recovery (Wróbel et al., 2025). This synergistic effect has been shown to significantly boost anthocyanin yields in red cabbage and cranberry, making acidified ethanol a safe choice for applications in food products (Franco-Londoño et al., 2025; Wróbel et al., 2025).

The crude extraction of anthocyanins from plant sources yields co-extractives, such as free sugars, organic acids, and proteins, which can interfere with downstream characterization and negatively impact the stability of the final product (Liu et al., 2020; Paul et al., 2023). Purification using macroporous resins (e.g., Amberlite XAD-7HP, AB-8, or NKA-9) is a crucial step to remove bulk impurities and enrich the total anthocyanin content. This adsorptive purification relies on hydrophobic interactions and hydrogen bonding to isolate the pigments, yielding a highly purified, crystalline extract suitable for commercial application. Amberlite XAD-7HP has been reported as a highly efficient resin (Heinonen et al., 2016; Liu et al., 2020; Tan et al., 2022).

The pigments are highly unstable under various environmental conditions, notably heat, pH, and light (Yücetepe et al., 2024). When comparing high-temperature treatments commonly used in food processing, anthocyanin degradation becomes highly pronounced at 70 °C, 80 °C, and 90 °C (Kaur et al., 2026; Wang & Xu, 2007). Studies indicate that as the temperature increases from ambient (25 °C) to 70 °C and 90 °C, the half-life of anthocyanin extracts from Roselle (*Hibiscus sabdariffa*) can decrease by 9.1 and 31 times, respectively (Saidji et al., 2024). The stability and color expression of anthocyanins are highly dependent on the pH of the surrounding medium (Yücetepe et al., 2024). Anthocyanins are highly stable and exhibit red color in acidic environments (pH 1 to 3) where they predominantly exist as flavylium cations (Gamage & Choo, 2023; Hu et al., 2014; Kruszewski & Boselli, 2024). They are highly susceptible to photodegradation upon exposure to light, including ultraviolet (UV) and standard visible light (Hu et al., 2014). Prolonged light exposure accelerates structural breakdown and color fading, whereas storage in dark conditions significantly enhances pigment retention and extends the overall half-life of the extracts (Saidji et al., 2024).

Despite the extensive literature review, several critical gaps remain for *Rosa chinensis Jacq*. First, while UAE-RSM optimisation of rose anthocyanins has been reported (Özgür & Çimen, 2018), studies employing food-grade, non-toxic solvent systems specifically acidified aqueous ethanol with organic acid combined with macroporous resin purification in a single integrated workflow remain limited. Second, the structural identity of the anthocyanins in *Rosa chinensis Jacq*. has not been unambiguously confirmed at a rigorous analytical standard. Prior studies have relied predominantly on HPLC-DAD retention time comparison or wide-target metabolomics profiling, which can only assign tentative identifications at MSI confidence Level 3 or below, due to the absence of MS/MS fragmentation evidence (Sumner et al., 2007). To our knowledge, no study has yet applied LC-ESI-MS/MS with collision-induced dissociation (CID) at multiple collision energies to confirm the identity of the principal anthocyanins of this species through diagnostic sequential neutral loss fragmentation, thereby achieving MSI Level 2 confidence. Third, comprehensive multi-parameter stability data encompassing thermal degradation kinetics, pH-dependent structural transitions, and photodegradation are rare for purified *Rosa chinensis* anthocyanins specifically.

To address these gaps, the present study reports: (i) the optimisation of UAE conditions for anthocyanin extraction from *Rosa chinensis Jacq*. petals using a food-grade acidified ethanol solvent system and a Box-Behnken design RSM; (ii) purification of the crude extract using Amberlite XAD-7HP macroporous resin; (iii) structural identification of the principal anthocyanins by LC-ESI-MS/MS in positive ion mode, with compound identities assigned at MSI confidence Level 2 based on accurate molecular ion masses and diagnostic MS/MS fragmentation cascades; and (iv) evaluation of thermal, pH, and light stability kinetics of the purified extract. The resulting integrated dataset provides both a rigorous phytochemical profile of *Rosa chinensis* anthocyanins and a validated, scalable extraction protocol suitable for functional food applications.

## 2 Materials and methods

### 2.1 Materials

Roses (*Rosa chinensis Jacq.* ‘Crimson Glory’ H.T.) were used in this study. The plant material was obtained from Anning, Kunming City, Yunnan Province, China. Analytical grade ethanol and citric acid were used for the preparation of extraction solvents.

### 2.2 Preparation of rose powder

Roses were subjected to complete drying in a hot air oven at 40 °C to preserve thermolabile compounds. The petals were ground into a fine powder using a mechanical grinder (Model 800C, Yongkang Hongtaiyang Electromechanical Co., Ltd., China) operating at 30,000 rpm and passed through a 40-mesh sieve to ensure a uniform particle size. The rose powder was stored in airtight bags at −80 °C.

### 2.3 Extraction procedure

The extraction was performed using an ultrasonic-assisted method (Zou et al., 2011). Precisely 1.0 g of the rose powder was weighed and mixed with a specified volume of extraction solvent. The mixture was subjected to extraction in an ultrasonic bath (KQ-300DE, Kunshan Ultrasonic Instrument Co., Ltd., China) operating at 100% power (300 W, 40 kHz). After sonication, the extract was centrifuged at 6000 rpm at 4 °C for 20 min to separate the solid residue. The supernatant was collected, and total anthocyanins of extracts were measured with pH differential method which was adopted from (Giusti & Wrolstad, 2001). All samples were filtered through a 0.22 μm syringe filter. All extraction experiments were conducted in triplicate (*n* = 3).

### 2.4 Single factor experiments

#### 2.4.1 Solvent screening

A screening of various solvent systems was conducted to identify the most effective medium for anthocyanin extraction by following the methodology of (Zou et al., 2011) with modification. The systems evaluated included: pure water; ethanol-water mixtures at 20%, 40%, 60%, 80%, and 100% (*v*/v) ethanol, ethanol combined with 2% (*w*/*v*) citric acid solution at ethanol:citric acid ratios of 90:10, 80:20, 70:30, 60:40, and 50:50 (*v*/v); and water combined with 2% (*w*/*v*) citric acid solution at water:citric acid ratios of 90:10, 80:20, 70:30, 60:40, and 50:50 (*v*/v). Precisely 1.0 g of rose powder was mixed with different solvents at a fixed solid-liquid ratio of 1:50 g mL^−1^. All extractions were performed in an ultrasonic bath at a controlled temperature of 40 °C for 45 min.

#### 2.4.2 S:L ratio optimization

Experiments were conducted to determine the optimal solid-to-liquid (S:L) ratio for anthocyanin extraction, testing five ratios: 1:40, 1:50, 1:60, 1:70, and 1:80 (g mL^−1^). The previously optimized extraction solvent was fixed, the temperature was 40 °C, and the extraction time was set at 45 min and processed according to the standard ultrasonic-assisted extraction procedure described in *Section 2.3*.

#### 2.4.3 Temperature optimization

Experiments were conducted to optimize the effect of temperature evaluated at five levels: 30, 40, 50, 60, and 70 °C. The extraction was performed using the previously identified optimal solvent and solid-to-liquid ratio and an extraction time of 45 min and processed following the protocol outlined in *Section 2.3*.

#### 2.4.4 Time optimization

A time-course study was conducted to evaluate the influence of extraction time on anthocyanin yield, testing seven time points: 10, 20, 30, 45, 60, 75, and 90 min. A 1.0 g sample of rose powder was combined with the previously determined optimal solvent and a temperature and processed following the protocol outlined in *Section 2.3*.

#### 2.4.5 Response surface methodology (RSM) design

A Box-Behnken Design (BBD) with three independent variables at three levels (−1, 0, +1) was used as shown in Table 1. The central point was replicated to estimate pure error. A total of 15 experimental runs were conducted. (See Table 2.)

**Table 1:** Design factors and coded levels for response surface experiments.

Independent variables	Design factors	Ranges and levels		
		Low value (−1)	0	High value (+1)
Temperature (°C)	A	30	40	50
Time (min)	B	10	20	30
Solid-to-liquid (*w*/*v*)	C	40	50	60

**Table 2:** The results of anthocyanin yield analysis.

Run	Variables			Responses
	*A* Temperature	*B* Time	*C* Solid-to-liquid	Y Anthocyanin yield (g kg^−1^)
1	0	+1	–1	32.5493
2	+1	+1	0	28.9648
3	–1	+1	0	33.6791
4	–1	0	–1	32.6853
5	0	0	0	34.5709
6	0	0	0	34.6559
7	+1	0	–1	29.0498
8	0	+1	+1	32.8721
9	–1	0	+1	33.1269
10	+1	0	+1	28.9988
11	0	–1	+1	31.0884
12	+1	–1	0	30.0691
13	–1	–1	0	31.3007
14	0	0	0	34.8258
15	0	–1	–1	32.1416

Note: Y = 34.68–1.71 A + 0.4332 B – 0.0425C – 0.8706 AB – 0.1232 AC + 0.3440 BCE – 2.44 A2–1.24 B2–1.28 C^2^.

### 2.5 Determination of anthocyanin content

The anthocyanin content in the extracts was determined using the pH differential method (Giusti & Wrolstad, 2001) with some modifications. In this method, the extract was appropriately diluted with potassium chloride (pH 1.0) and sodium acetate (pH 4.5) buffers, respectively. The absorbance of each solution was then measured at 520 nm (λ_max_) and 700 nm (for haze correction) against a blank using a Unico UV-2802 spectrophotometer (Unico Systems, Inc., USA). Results are expressed as g kg^−1^ DW of cyanidin-3,5-*O*-Diglucoside equivalents. The anthocyanin content was calculated using the following formula:

(1) Anthocyanin content (mg L^−1^) = (ΔA × MW × DF × 1000)/(ε × L)

Whereas ΔA = [(A_520_ - A_700_) _pH1.0_ - (A_520_ - A_700_) _pH4.5_].

MW (molecular weight of cyanidin-3,5-*O*-Diglucoside) = 611.16 g mol^−1^.

DF = dilution factor.

ε (molar extinction coefficient) = 29,500 L mol^−1^ cm^−1^.

L = 1 cm (path length of cuvette).

### 2.6 Purification of anthocyanins

The crude anthocyanin extract was purified using Amberlite XAD-7HP macroporous resin to remove sugars, organic acids, and other water-soluble impurities according to the method of (Chen et al., 2016) with some modifications. Briefly, the Amberlite XAD-7HP resin (100 g) was activated by soaking in 95% ethanol for 12 − 16 h to swell the beads, followed by washing with distilled water to remove potential organic contaminants. After that, the resin was equilibrated with 1% citric acid solution and wet packed into a glass chromatography column (2.6 cm × 50 cm). The crude ethanolic extract (approximately 1 L) was concentrated to 400 mL to remove ethanol, and was loaded onto the column at a controlled flow rate of 1.0 mL min^−1^. The column was washed with 3–4 BV h^−1^ of distilled water. The adsorbed anthocyanins were then eluted using 1–2 BV h^−1^ of acidified ethanol (70% Ethanol (*v*/v) + 1% Citric acid (*w*/*v*)). The anthocyanin-rich ethanolic fraction was collected and concentrated using a rotary evaporator initially at 40 °C and then freeze dried to obtain the purified anthocyanin powder.

The purification efficiency was calculated using the following equations:

(2) **Recovery (%)** = (TAC_P_ × W_P_)/ (TAC_E_ × W_RP_) × 100

where TAC_P_ is the Total Anthocyanins in Purified Extract, TAC_E_ is Total Anthocyanins in Crude Extract, W_P_ is weight of purified anthocyanin in grams, W_RP_ is weight of rose powder in grams.

(3) **Enrichment Factor** = TAC_P_/TAC_E_

TAC_P_ is Total Anthocyanin content after purification (g kg^−1^), TAC_E_ is Total Anthocyanin content before purification (g kg^−1^).

### 2.7 Determination of antioxidant activity

#### 2.7.1 DPPH radical scavenging assay

The antioxidant capacity was determined using the DPPH (2,2-diphenyl-1-picrylhydrazyl) radical scavenging assay as described by (Li et al., 2019) with modifications. Briefly, 0.1 mL of the rose petal extract at various concentrations was mixed with 3.9 mL of a 0.1 mM DPPH ethanolic solution. The absorbance was measured at 517 nm using a Unico UV-2802 spectrophotometer (Unico Systems, Inc., USA). Trolox was used as the standard, and the radical scavenging activity was expressed as IC_50_. The final antioxidant capacity was reported as mmol TE kg^−1^ DW.

The percentage of inhibition was calculated according to the following equation:

(4) Inhibition (%) = [(A_0_ – A_S_) / A_0_] × 100

where A_0_ is the absorbance of the control and A_S_ is the absorbance of the sample.

#### 2.7.2 ABTS radical scavenging assay

The ABTS (2,2′-azino-bis(3-ethylbenzothiazoline-6-sulfonic acid)) assay was performed according to the method of (Xu et al., 2017). The ABTS radical cation (ABTS·^+^) stock solution was generated by reacting 7 mM ABTS with 2.45 mM potassium persulfate (1:1, *v*/v) and incubating the mixture in the dark for 12–16 h. Before use, the stock solution was diluted with ethanol to an absorbance of 0.70 ± 0.02 at 734 nm. An aliquot of 0.1 mL of sample was mixed with 3.9 mL of the diluted ABTS·^+^ solution. After 6 min of incubation in the dark, the absorbance was measured at 734 nm and ethanol was used as blank. Results were expressed as mmol TE kg^−1^ DW. The percentage of inhibition was calculated according to eq. 4.

### 2.8 Stability studies

#### 2.8.1 Thermal stability

The effect of different temperatures on purified anthocyanin powder was determined following the methodology given by (Shiau et al., 2025; Swer & Chauhan, 2019) with modifications. Purified anthocyanin powder was dissolved in acidified distilled water (pH 3.0) to achieve an initial absorbance of approximately 1.0 at 520 nm. Equal volume of the solution were divided into clean screw capped amber colored bottles and were subjected to heating at 70 °C, 80 °C and 90 °C in water bath for 0, 30, 60, 90, 120, 150, and 180 min, then sample was taken out and immediately cooled to room temperature and the absorbance was read at 520 nm in a Unico UV-2802 spectrophotometer (Unico Systems, Inc., USA), using the same solvent as blank.

The degradation kinetics of anthocyanins under thermal treatment was analyzed based on a first-order reaction model:

(5) ln (A_t_/ A_0_) = −k × t

where A_0_ represents the initial absorbance, A_t_ denotes the absorbance remaining after heating for t hours at a specific temperature, and k is the rate constant of the degradation reaction.

The half-life (t_1/2_) defined as the time required for the absorbance to reduce by half, was determined using the following expression:

(6) t_1/2_ = ln (2)/k

The activation energy was determined following the Arrhenius equation:

(7) *lnk* = −E_a_/RT + *ln*A

where E_a_ is the activation energy, T is the heating temperature, R is the gas constant, and A is the frequency factor.

#### 2.8.2 pH stability

The stability of purified anthocyanin powder was evaluated at different pH values by following the method given by (Hu et al., 2014) with modifications. The solvents used for buffer solutions (pH 2, 3, 4, 5, 6, 7, 8, and 9) were citrate-phosphate buffers. A 5 mM (2.246 g L^−1^) anthocyanin solution was prepared in acidified distilled water (pH 3.0). 1 mL anthocyanin solution was added to 20 mL pH buffer solution, and the absorbance of each sample was subsequently measured at wavelength of 190–700 nm after dissolving the extract concentrate for 1 h in the buffer solution. All the samples were stored in dark 4 °C refrigerator. The pigment retention was calculated based on the absorbance at the maximum wavelength (λ_max_) specific to each pH.

Further, percent pigment retention was calculated on daily basis for 10 d as percentage of the absorbance on initial day.

(8) Pigment Retention (%) = Absorbance after time T/initial absorbance × 100

#### 2.8.3 Light stability

Effect of light on purified anthocyanin powder was evaluated by exposing the anthocyanin solution (pH 3.0) directly beneath a 40 W lamp at a distance of 30 cm, by following the method of (Hu et al., 2014) with modifications. The solutions were divided into two parts. One part was transferred to clean screw capped amber colored vial whereas the other part was transferred to clean screw capped colorless glass vial. The absorbance was read in a Unico UV-2802 spectrophotometer (Unico Systems, Inc., USA), at 520 nm using the same solvent as blank. Percent pigment retention was calculated on daily basis for 10 days as percentage of the absorbance on initial day according to Eq. 8.

### 2.9 HPLC analysis and identification of anthocyanins

The identification of anthocyanins were performed using an Agilent 1260 Infinity II HPLC system equipped with a diode array detector (DAD) (Agilent Technologies, USA) by following the method of (Ge & Ma, 2013; Heinonen et al., 2016) with some modifications. Separation was achieved on an Agilent HC-C18(2) column (250 × 4.6 mm, 5 μm) maintained at ambient temperature (25 °C).

The mobile phase consisted of 1% formic acid in water (A) and acetonitrile (B). The elution was performed using the following gradient program at a flow rate of 1.0 mL min^−1^: 0–5 min, 5–10% B; 5–15 min, 10–20% B; 15–25 min, 20–30% B; 25–30 min, 30–50% B; followed by a re-equilibration step of 35–40 min at 5% B. The injection volume was 20 μL.

### 2.10 LC-ESI-MS/MS structural identification

Structural identification was performed on an Agilent 6545 series LC-Q-TOF system (Agilent Technologies, Santa Clara, CA, USA) coupled to an electrospray ionisation (ESI) source, using the C18 column (4.6 × 250 mm, 5 μm) by following the method of (Ge & Ma, 2013) with modifications. The purified anthocyanin powder (1.0 mg) was dissolved in 1 mL of 50% aqueous methanol containing 0.1% formic acid (*v*/v) to yield a stock solution of 1 mg/mL. The solution was filtered through a 0.22 μm PTFE membrane and transferred into amber HPLC vials.

Mobile phase A was 0.1% formic acid in ultrapure water and mobile phase B was 0.1% formic acid in acetonitrile. The gradient program was: 0–10 min, 5% B, 10–30 min, 5–20% B; 30–60 min, 20–50% B; 60–70 min, 50–5% B; 70–80 min, 5% B (re-equilibration). The flow rate was 0.5 mL min^−1^, and the column temperature was 35 °C. Mass spectrometric detection was performed in positive electrospray ionisation (ESI^+^) mode. In-source fragmentation voltage (Frag) was set at 135.0 V. Full-scan mass spectra were acquired over the range *m*/*z* 100–1000. For structural characterization, product ion (MS/MS) spectra were acquired by isolating each molecular ion [M + H]^+^ and subjecting it to collision-induced dissociation (CID) at multiple collision energies (20, 30, and 40 eV) using nitrogen as the collision gas. Data acquisition and processing were performed using Agilent MassHunter Workstation software (version B.07.00).

Compound identification was performed by comparing the observed molecular ions [M + H]^+^ and characteristic MS/MS product ions with published fragmentation data for anthocyanins in *Rosa* spp. (Barnes & Schug, 2011; Wan et al., 2019). Identification confidence was assigned according to the Metabolomics Standards Initiative (MSI) reporting guidelines (Sumner et al., 2007). In the present study, all anthocyanin identifications were assigned at MSI confidence Level 2.

### 2.11 Statistical analysis

The experimental design for the response surface methodology was generated using Design-Expert® software (Version 13, Stat-Ease Inc., Minneapolis, MN, USA), employing a Box-Behnken Design (BBD) with three factors at three levels. Single factor optimization graphs were generated using Origin® software (Version 2025b, Origin Lab Corporation, Northampton, MA, USA). Data were expressed as mean ± standard deviation of triplicate determinations, and differences among means were analyzed using one-way ANOVA followed by Duncan's multiple range test, with significance indicated by different letters (*p* < 0.05).

## 3 Results & discussion

### 3.1 Effect of solvent composition

**Fig. 1:**
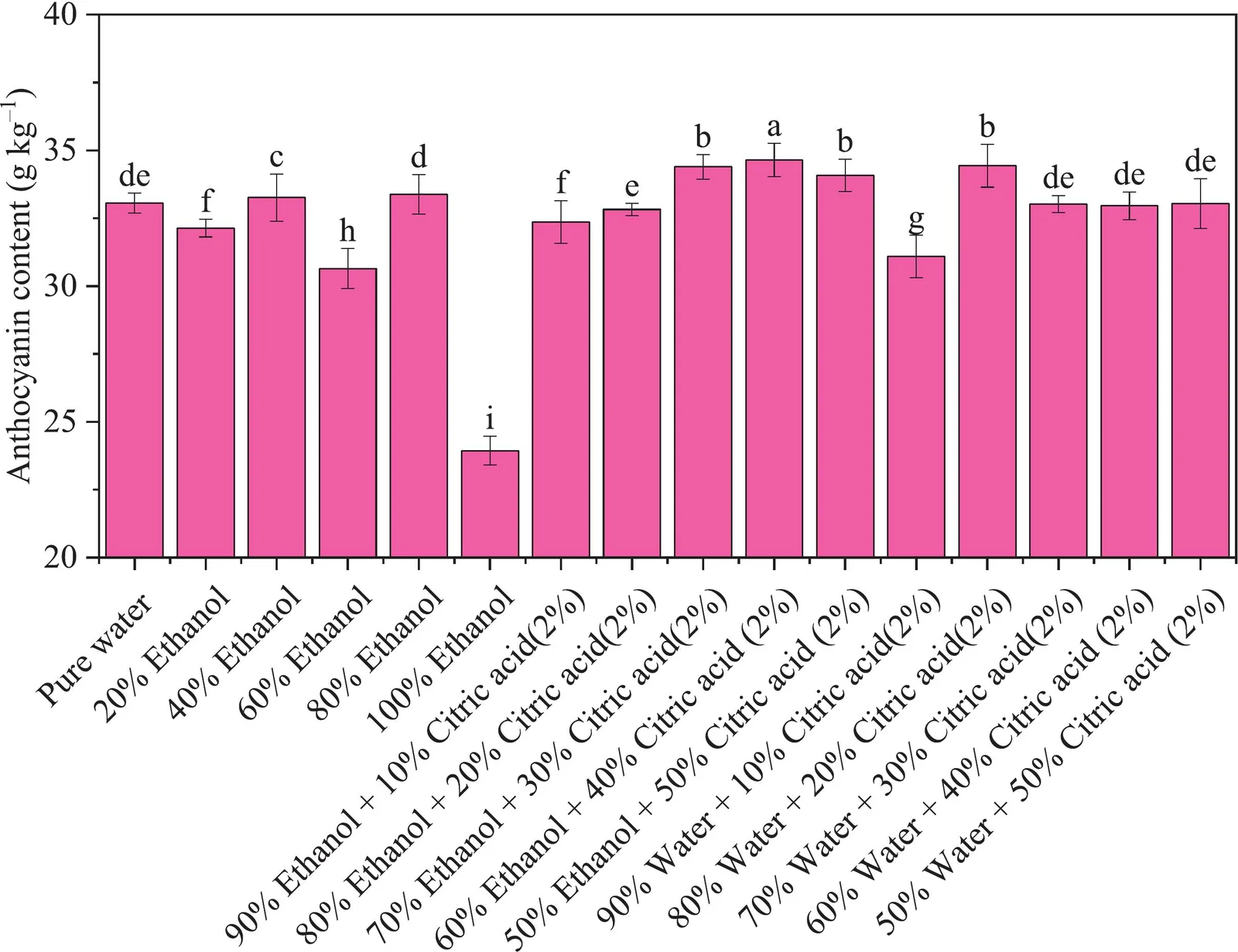
Effect of solvent composition on anthocyanin yield from *Rosa chinensis Jacq*. Data represent mean ± SD (*n* = 3). Bars with different lowercase letters indicate significant differences (*p* < 0.05, Duncan's test).

The composition of the extraction solvent significantly influenced anthocyanin yield from rose petals (Fig. 1). Among non-acidified solvents, a mixture of ethanol and pure water at a volume ratio of 80:20 yielded the highest anthocyanin content (33.37 g kg^−1^ DW). The addition of citric acid shifted the optimum solvent ratio; the highest overall yield (34.64 g kg^−1^ DW) was achieved with a mixture of ethanol and a 2% (*w*/*v*) citric acid solution at a volume ratio of 60:40. This indicates that a moderate ethanol concentration combined with an acidic environment provides the optimal balance for extraction. The addition of 2% citric acid enhanced extraction yields across all solvent combinations due to synergistic role of both ethanol concentration and acidic pH. Common solvents in research include high-concentration methanol (e.g., 70% acidified with HCl or formic acid), which often yields the highest extraction efficiency due to its strong polarity and cell wall disruption capabilities (Chandra Singh et al., 2022; Silva et al., 2017; Taghavi et al., 2022). Similarly, acetone-water mixtures (typically 60–70%) are also highly effective (Kuppusamy et al., 2023). However, their toxicity and residual concerns render them unsuitable for food-grade applications. Alternatively, water alone is safe but inefficient, often yielding significantly less anthocyanins than organic solvents due to poor penetration and higher co-extraction of polar impurities (Silva et al., 2017).

The yield enhancement with acidified ethanol over the non-acidified underscores the critical role of pH modulation. This synergistic effect aligns with findings in other matrices, where acidified ethanol significantly boosted anthocyanin recovery (Silva et al., 2017). Furthermore, the use of a weak organic acid like citric acid avoids the potential for hydrolytic degradation associated with mineral acids, makes the extract suitable for functional food applications (Khoo et al., 2017; Utomo et al., 2020).

### 3.2 Effect of solid liquid ratio on anthocyanin extraction rate

**Fig. 2:**
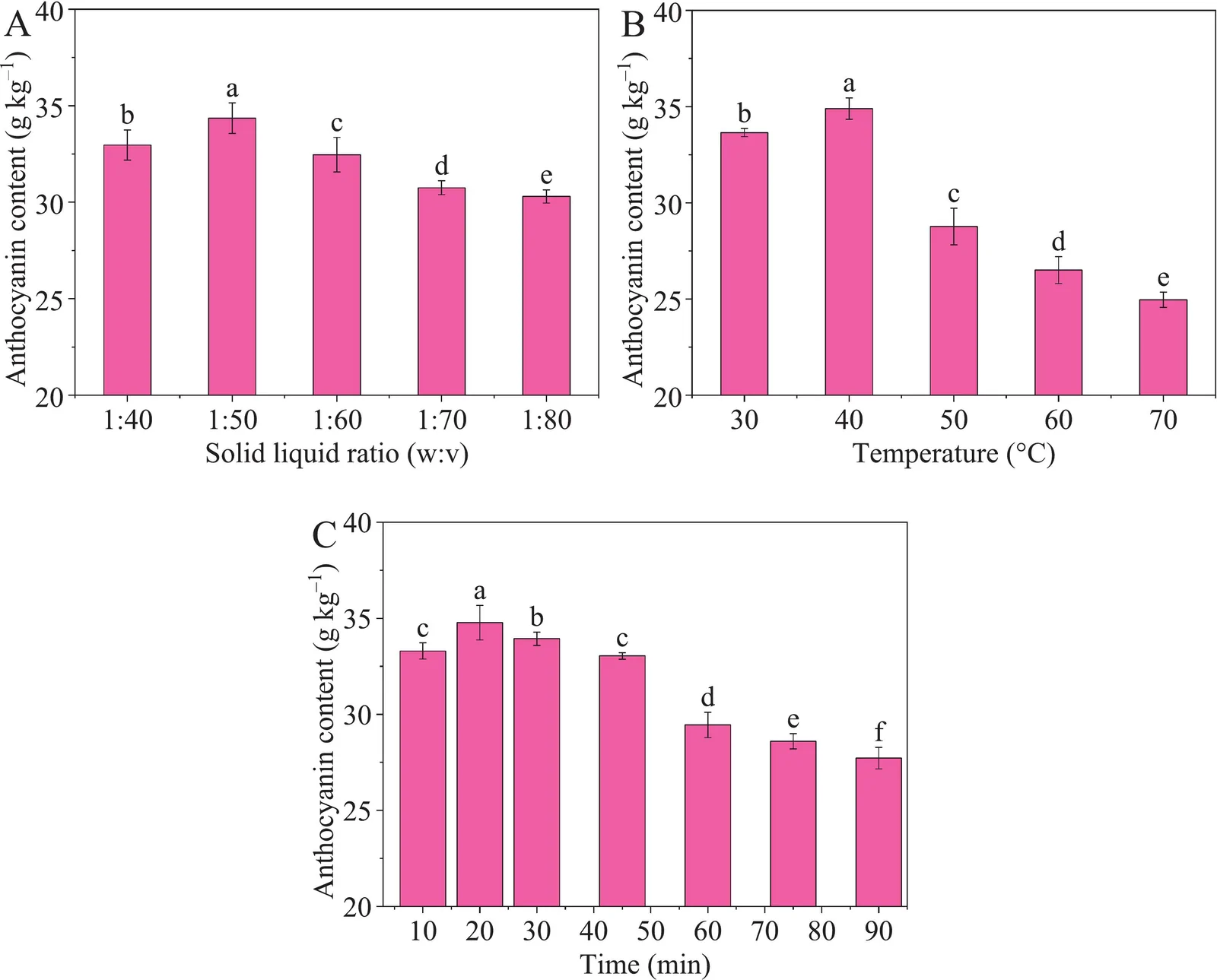
Effect of solid-to-liquid (S:L) ratio (A), temperature (B) and time (C) on anthocyanin yield from *Rosa chinensis Jacq*. Data represent mean ± SD (*n* = 3). Means with different lowercase letters are significantly different (*p* < 0.05, Duncan's test).

The solid-to-liquid (S/L) ratio has significant impact on the extraction efficiency of anthocyanins from rose petals. As illustrated in Fig. 2A, the anthocyanin yield varied considerably across the tested S:L ratios ranging from 1:40 to 1:80 g mL^−1^. The maximum anthocyanin content of 34.3 g kg^−1^ DW was achieved at a S:L ratio of 1:50 g mL^−1^. The observed pattern suggests that an optimal balance exists between the solid matrix and solvent volume recovery (Silva et al., 2017). Based on these results, the S:Lratio of 1:50 g mL^−1^ was therefore fixed at the center point for the subsequent RSM design to optimize the interactive effects of other key variables.

### 3.3 Effect of temperature on anthocyanin extraction rate

Extraction temperature demonstrated a major influence on anthocyanin yield from rose petals. The results shows a clear trend of decreasing anthocyanin content with increasing temperature beyond a certain point as shown in Fig. 2B. At a fixed extraction time of 45 min, the maximum anthocyanin yield of 34.89 g kg^−1^ DW was obtained at 40 °C. Yields at 30 °C and 40 °C were similar. Further increase in temperature to 50 °C, 60 °C, and 70 °C resulted in lower anthocyanin contents of 28.7, 26.5 and 24.9 g kg^−1^ DW, respectively. This consistent decline in extraction efficiency at higher temperatures suggests thermal degradation of anthocyanins, which are known to be heat-sensitive compounds. Therefore, prolonged exposure to high temperatures should be avoided to reduce the chalcone formation and losses of anthocyanin stability (Arruda et al., 2021). The inverse relationship between temperature and anthocyanin yield indicates that mild temperatures are preferable for maximizing anthocyanin extraction from rose petals while preserving their structural integrity.

### 3.4 Effect of time on anthocyanin extraction rate

The extraction time demonstrated a notable, non-linear influence on anthocyanin yield. As illustrated in Fig. 2C, the anthocyanin content reached a maximum of 34.76 g kg^−1^ DW at 20 min and 40 °C, establishing this as the optimal duration, which is similar to the extraction from Grape peel, i.e., 24.5 min (Ghafoor et al., 2011). Shorter extraction (10 min) resulted in incomplete extraction, whereas longer times led to a gradual decline in yield. This pattern suggests that while sufficient time is required to reach equilibrium concentration, prolonged exposure may induce anthocyanin degradation or facilitate the co-extraction of compounds that promote pigment decomposition (Arruda et al., 2021).

### 3.5 Results of response surface experiment

#### 3.5.1 Model interpretation

The fitness and significance of the developed quadratic model for anthocyanin yield were rigorously evaluated by analysis of variance (ANOVA) (Table 3). The model was highly significant (*p* = 0.0003) with an F-value of 45.86, indicating it is statistically robust and capable of predicting the extraction process. The coefficient of determination (R^2^ = 0.9880) confirms that the model explains 98.80% of the total variation in the response. Furthermore, the non-significant lack of fit (*p* = 0.0704) demonstrates that the model adequately fits the experimental data.

**Table 3:** Analysis of variance (ANOVA) for regression equations.

Source	Sum of Squares	DF	Mean Square	F-Value	*P*-Value
Model	58.53	9	6.50	45.86	0.0003***
A-Temperature	23.49	1	23.49	165.68	< 0.0001***
B-Time	1.50	1	1.50	10.59	0.0226*
C-S:L ratio	0.014	1	0.0144	0.1018	0.7626
AB	3.03	1	3.03	21.38	0.0057**
AC	0.0607	1	0.0607	0.4279	0.5419
BC	0.4734	1	0.4734	3.34	0.1272
A^2^	21.97	1	21.97	154.92	< 0.0001***
B^2^	5.69	1	5.69	40.14	0.0014***
C^2^	6.05	1	6.05	42.65	0.0013***
Residual	0.7090	5	0.1418		
Lack of Fit	0.6754	3	0.2251	13.37	0.0704
Pure Error	0.0337	2	0.0168		
Cor Total	59.24	14			
Std. Dev.	0.3766				
Mean	32.04				
C.V. %	1.18				
*R*^2^ = 0.9880	*R*^*2*^*Adj* = 0.9665	Coefficient of variation = 18.96%			

Note: *, Significant (*p* < 0.05); **, Significant (*p* < 0.01); ***, Significant (*p* < 0.001).

The ANOVA results delineate the individual and interactive effects of the independent variables. The linear term of temperature (A) was extremely significant (*p* < 0.0001), while the linear term of time (B) was also significant (*p* = 0.0226), with temperature exhibiting the more substantial effect on anthocyanin yield. In contrast, the linear effect of the solid-to-liquid (S:L) ratio (C) was not significant (*p* = 0.7626). Crucially, two-factor interaction terms (AC, BC) were statistically non-significant (*p* > 0.05). However, the quadratic effects of all three factors (A^2^, B^2^, and C^2^), were highly significant (*p* < 0.01), confirming pronounced curvilinear relationships for all factors. The model identifies extraction temperature and time as the dominant factors. The final empirical relationship in terms of coded factors is expressed by the following equation:

Anthocyanin Content (g kg^−1^ DW) = Y = 34.68–1.71 A + 0.4332 B – 0.0425C – 0.8706 AB – 0.1232 AC + 0.3440 BCE – 2.44 A^2^–1.24 B^2^–1.28 C^2^.

#### 3.5.2 Interpretation of response surface and contour plots

**Fig. 3:**
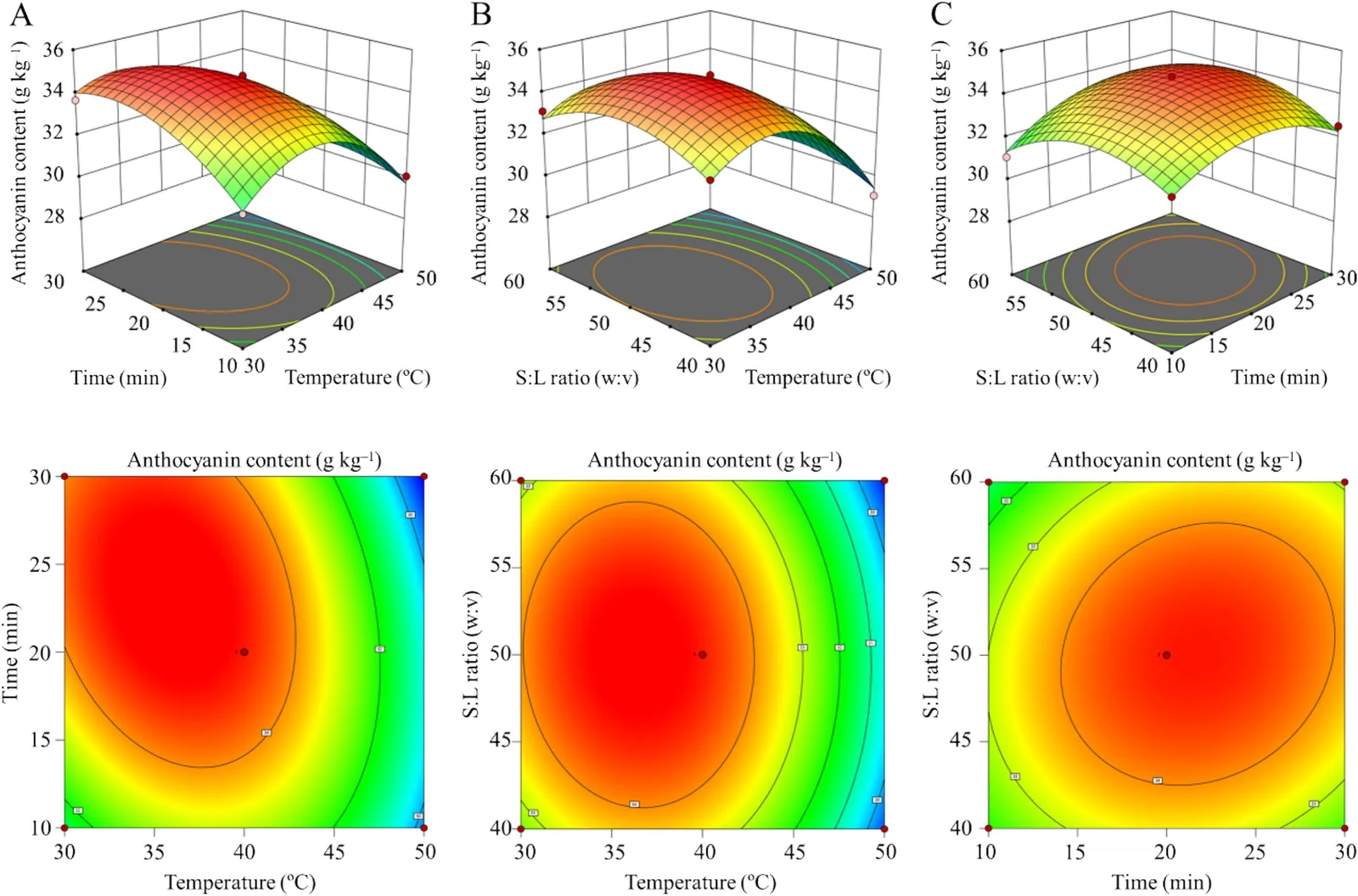
3D response surface plot showing the interactive effect of extraction temperature and time (A), extraction temperature and solid-to-liquid ratio (B), extraction time and solid-to-liquid ratio (C) on anthocyanin yield from edible roses.

The response surface (Fig. 3A) exhibits a dome-shaped curvature, clearly illustrating the strong individual and quadratic effects of temperature and time. The anthocyanin yield peaks at moderate levels of both factors. The corresponding contour plot shows a series of elliptical contours centered around the optimum at approximately 40 °C and 20 min, with the major axis of the ellipses tilted relative to the temperature and time coordinate axes. This tilted, elliptical pattern provides graphical confirmation of the significant interactive effect between temperature and time (AB, *p* = 0.0057), as a non-significant interaction would instead produce contours that are circular/symmetric and aligned parallel to the axes.

The response surface (Fig. 3B) reveals that yield is sensitive to temperature changes across all S:L ratios, as seen by the steep slope of the surface. In contrast, the surface is relatively flat along the S:L ratio axis, consistent with its non-significant linear effect. The contour plot displays a broad, flat optimum region along the S:L ratio axis, indicating that the process is robust to variations in this factor around its center point, visually supports the insignificant AC interaction term (*p* = 0.5419).

The response surface and contour plot (Fig. 3C) for time and S:L ratio show a clear peak. The contours are approximately circular, centered at around 20 min and a 1:50 g mL^−1^ ratio. This symmetrical pattern reinforces the statistical conclusion that the interaction between time and S:L ratio (BC) is not significant (*p* = 0.1272). The significant quadratic effect of the S:L ratio (C^2^) is evidenced by the closed contours in the response plots, confirming a well-defined optimum within the experimental domain. Elevated temperatures enhance initial mass transfer, prolonged exposure beyond an optimum accelerates degradation via hydrolysis and oxidation (Arruda et al., 2021). The optimum yield was achieved rapidly within 20 min, which can be attributed to the mechanism of ultrasound-assisted extraction. Ultrasound cavitation accelerates the extraction process compared to conventional soaking (Franco-Londoño et al., 2025; Lucas-González et al., 2025).

The final optimized UAE process achieved a high yield of anthocyanins. This outcome is notably superior to yields typically reported in studies using conventional solvent extraction or enzymatic methods on rose petals (Ribeiro et al., 2024).

#### 3.5.3 Results of verification test

A confirmation experiment was conducted to validate the predictive accuracy of the quadratic model under the model-predicted optimal conditions: extraction temperature of 36.3 °C, time of 23.9 min, and solid-to-liquid ratio of 1:49.4 g mL^−1^, using a solvent system of 60% (*v*/v) ethanol and 40% (*v*/v) aqueous citric acid (2% *w*/*v*). The mean experimental anthocyanin yield was 34.99 ± 0.48 g kg^−1^ DW (mean ± SD). This value was compared to the model-predicted yield (35.076 g kg^−1^), resulting in a relative error of less than 1%.

### 3.6 Efficiency of anthocyanin purification

The crude extract obtained from *Rosa chinensis* contains high levels of soluble sugars and organic acids, which can interfere with downstream characterization and reduce the stability of the anthocyanins during storage (Heinonen et al., 2016). The purification process using Amberlite XAD-7HP resin significantly improved the quality of the extract and results are summarized in Table 5. The purification process achieved a high anthocyanin recovery of 79.72%, indicating that the XAD-7HP resin has a strong adsorption capacity for rose anthocyanins. The total anthocyanin content (TAC) increased which corresponds to a 4.06-fold enrichment factor, demonstrating the effective removal of bulk impurities. This robust recovery aligns with previous studies that have established XAD-7HP as a highly efficient macroporous resin for the separation and purification of anthocyanins from various botanical sources, due to its favorable polarity and high surface area (Chen et al., 2016; Heinonen et al., 2016).

### 3.7 Antioxidant activity of the optimized extract

The antioxidant potential of the *Rosa chinensis* extract was evaluated using both DPPH and ABTS assays. Extract exhibited an IC_50_ value of 351.95 mg L^−1^and 292.72 mg L^−1^ for DPPH and ABTS assay respectively, while the standard Trolox showed an IC_50_ of 179.44 mg L^−1^ and 106.65 mg L^−1^ for DPPH and ABTS assay respectively. Based on these values, the radical scavenging capacity of the extract was determined to be 2036.39 and 1455.15 mmol TE kg^−1^ DW for DPPH and ABTS assay respectively.

These results (Table 5) indicate that the optimized ethanol-citric acid extraction method effectively recovers bioactive anthocyanins with significant antioxidant capacity. The slightly higher activity observed in the DPPH assay compared to ABTS may be attributed to the specific selectivity of the radicals toward the different phenolic structures present in the rose petals. This free radical scavenging capacity is highly comparable to the DPPH IC_50_ of 302.89 mg L^−1^ reported for pomegranate peel anthocyanin extracts (Zahed & Kenari, 2025), indicating a similarly potent antioxidant profile in complex plant extracts. However, the extract's scavenging activity is lower than that of highly purified or enzymatically acylated rose anthocyanins, which have exhibited exceptional DPPH IC_50_ values of 22.917 mg L^−1^ and 4.451 mg L^−1^, respectively (Li et al., 2023a). It is also less potent than natural deep eutectic solvent (NADES) extracts of dried rose flowers, which achieved a DPPH IC_50_ of 2.1 mg L^−1^ (Prakash et al., 2024).

Consistent with the DPPH trends, this ABTS scavenging capacity demonstrates moderate potency when contrasted with highly purified natural rose anthocyanins (19.443 mg L^−1^) and acylated rose anthocyanins (7.76 mg L^−1^) (Li et al., 2023a).

### 3.8 Stability studies

#### 3.8.1 Thermal degradation kinetics

**Fig. 4:**
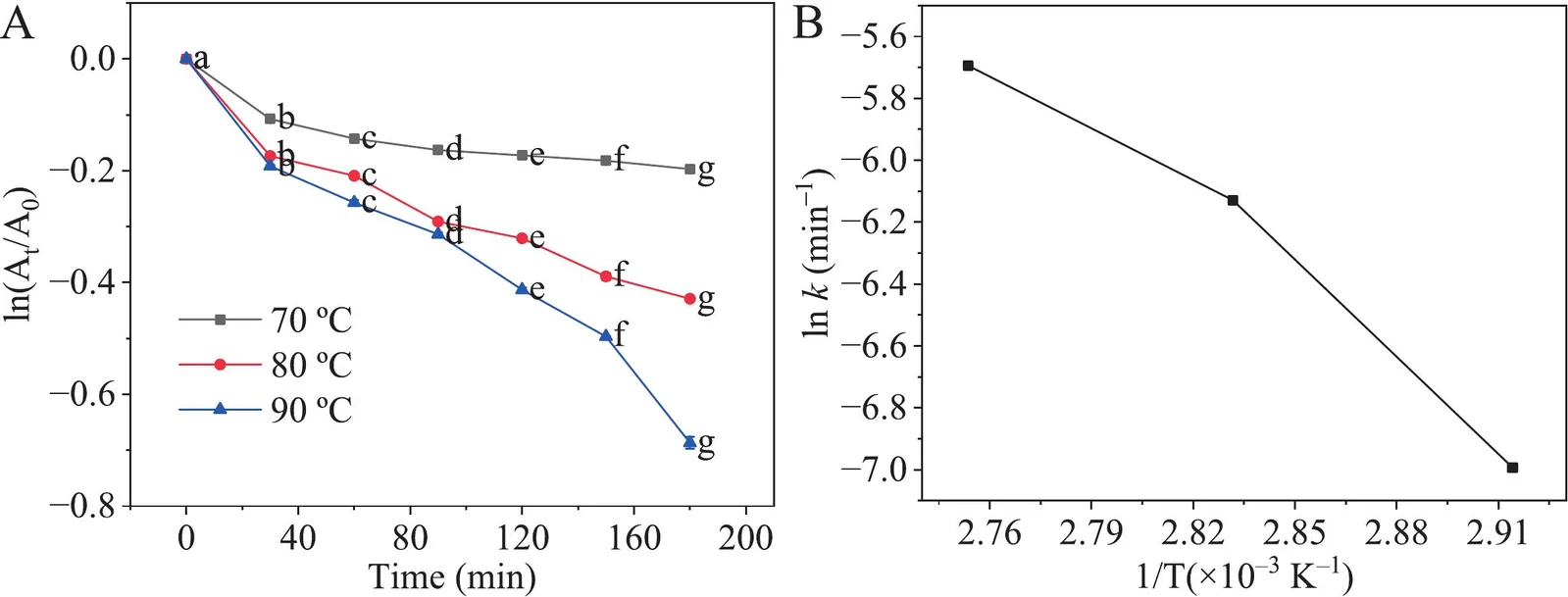
First-order thermal degradation kinetics of *Rosa chinensis Jacq.* anthocyanin extracts at 70 °C, 80 °C, and 90 °C. Data represent mean ± SD (*n* = 3) (A), The Arrhenius plot for degradation of anthocyanins during heating (B).

The thermal stability of the purified *Rosa chinensis* anthocyanins was evaluated at 70 °C, 80 °C, and 90 °C. The degradation of anthocyanins followed first-order reaction kinetics (Wang & Xu, 2007), as indicated by the linear relationship between the natural logarithm of the retention ratio ln(*C*_*t*_*/C*_*0*_) and heating time as shown in Fig. 4A.

The kinetic parameters, including the degradation rate constant (*k*) and half-life (*t*_*1/2*_), are summarized in Table 4. Temperature had a profound effect on the stability of the pigment. The degradation rate (*k*) increased substantially with rising temperature. Consequently, the half-life (*t*_*1/2*_*)* decreased from approximately 12.6 h at 70 °C to just 3.4 h at 90 °C. The comparatively lower R^2^ value at 70 °C (0.778) relative to 80 °C and 90 °C (0.936 and 0.966, respectively) is attributable to the limited extent of pigment loss at this milder temperature within the tested time frame, which reduces the signal-to-noise ratio of the degradation curve and increases the relative influence of measurement variability on the linear fit, rather than indicating a departure from first-order degradation behavior.

**Table 4:** Degradation kinetic parameters of *Rosa chinensis Jacq*. anthocyanin extracts under varying temperature, pH, and light conditions.

**Influencing factors**		**Degradation rate constant (k) (h**^**−1**^**)**	**Correlation coefficient (R**^**2**^**)**	**Half-life (t**_**1/2**_**) (h)**
**Temperature (°C)**	70	0.0551 ± 0.0013	0.778	12.59 ± 0.30
	80	0.1308 ± 0.0026	0.936	5.30 ± 0.11
	90	0.2019 ± 0.0024	0.966	3.43 ± 0.04
	Activation Energy E_a_	67.50 ± 0.63 kJ mol^−1^		
**pH**	pH 2	0.00255 ± 0.00034	0.953	275.50 ± 38.22
	pH 3	0.00664 ± 0.00087	0.978	105.51 ± 13.91
	pH 4	0.02640 ± 0.00248	0.993	26.41 ± 2.62
	pH 5	0.04437 ± 0.01134	0.997	16.46 ± 4.92
	pH 6	0.02806 ± 0.00528	0.943	25.36 ± 5.32
	pH 7	0.07381 ± 0.00307	0.654	9.40 ± 0.40
	pH 8	0.15827 ± 0.00152	0.506	4.38 ± 0.04
	pH 9	0.08989 ± 0.00414	0.576	7.72 ± 0.36
**Light**	Dark	0.0173 ± 0.0015	0.928	40.25 ± 3.52
	Light	0.0354 ± 0.0011	0.962	19.58 ± 0.63

Note: Values are presented as Mean ± SD (*n* = 3).

**Table 5:** Purification efficiency, DPPH and ABTS radical scavenging activity of anthocyanin extract from *Rosa chinensis* Jacq.

Purification				Antioxidant activity			
				DPPH		ABTS	
Crude extract TAC (g kg^−1^)	Purified extract TAC (g kg^−1^)	Recovery %	Enrichment factor	IC_50_ (mg L^−1^)	TEAC (mmol TE kg^−1^)	IC_50_ (mg L^−1^)	TEAC (mmol TE kg^−1^)
34.46 ± 0.86	139.89 ± 3.69	79.72 ± 3.98	4.06 ± 0.20	351.95 ± 6.83	2036.39 ± 51.14	292.72 ± 7.38	1455.15 ± 32.41

Note: Values are presented as mean ± SD (*n* = 3). TAC: total anthocyanin content; TEAC: Trolox equivalent antioxidant capacity.

Notably, at 70 °C, the extract retained >85% of its initial color intensity after 2 h of heating. This relatively high stability at pasteurization-relevant temperatures suggests that the purified pigment is suitable for thermal processing conditions commonly used in the food industry (Gamage & Choo, 2023).

The temperature dependence of the degradation rate was further analyzed using the Arrhenius equation (Fig. 4B). The calculated activation energy (*E*_*a*_) was 67.50 kJ mol^−1^. This high activation energy indicates that the purified anthocyanins are relatively sensitive to temperature elevation, requiring significant energy to initiate the degradation of the flavylium cation structure (Wang & Xu, 2007). Our results aligns with the kinetic models established for other plant sources like blackberry juice and sour cherry. When compared to literature, purified rose anthocyanins exhibit superior thermal stability to blackberry juice, which reported a shorter half-life of 8.8 h at the same 70 °C temperature (Wang & Xu, 2007). The high retention of >85% of initial color after 2 h at 70 °C confirms that the extract is suitable for pasteurization-relevant conditions, similar to encapsulated red cabbage anthocyanins designed for mild yogurt pasteurization at 70 °C (Kaur et al., 2026). Furthermore, the activation energy (E_a_) falls within the expected thermodynamic range for anthocyanin degradation. For comparison, blackberry juice concentrate exhibits an (Ea) of 65.06 kJ mol^−1^ (Wang & Xu, 2007), and acerola pulp exhibits an (Ea) of 68.04 kJ mol^−1^ (Oancea, 2021). This high (Ea)value corroborates the literature consensus that anthocyanin breakdown is highly susceptible to temperature elevations, requiring substantial energy to initiate the endothermic opening of the pyrylium ring (Özgür & Çimen, 2018).

#### 3.8.2 pH degradation kinetics

**Fig. 5:**
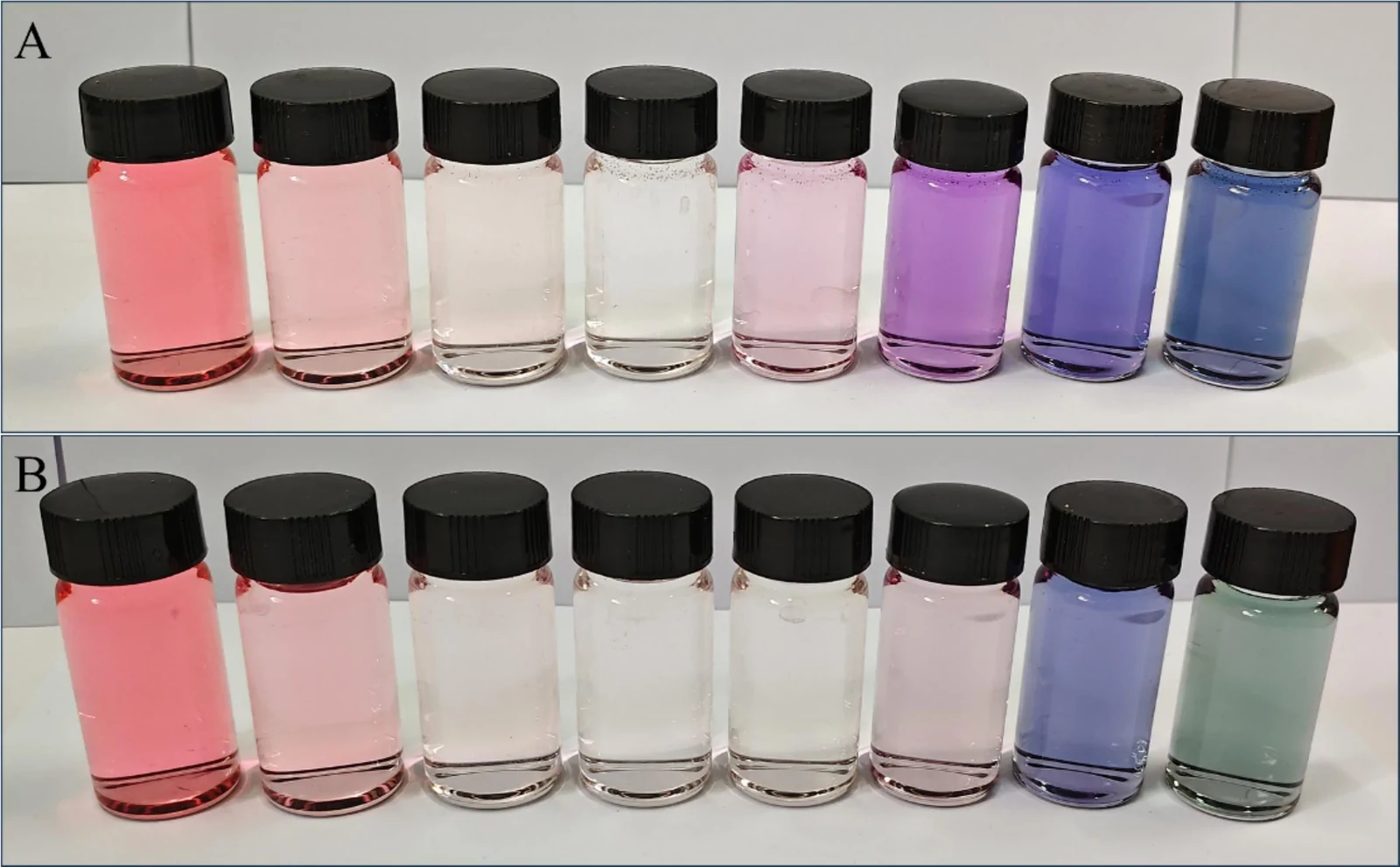
Visual color changes of purified *Rosa chinensis* Jacq. anthocyanin extract at pH 2–9 (left to right). **(A)** Immediately after pH adjustment; **(B)** After 1 h of equilibration at each pH. The color progression reflects the structural transformations of anthocyanins: bright red/pink at acidic pH (2–3) corresponding to the dominant flavylium cation form, colorless at pH 4–5 due to formation of the carbinol pseudobase, violet/purple at pH 6–7 indicating quinoidal base formation, and blue-green at pH 8–9 reflecting further deprotonation and onset of degradation. (For interpretation of the references to color in this figure legend, the reader is referred to the web version of this article.)

**Fig. 6:**
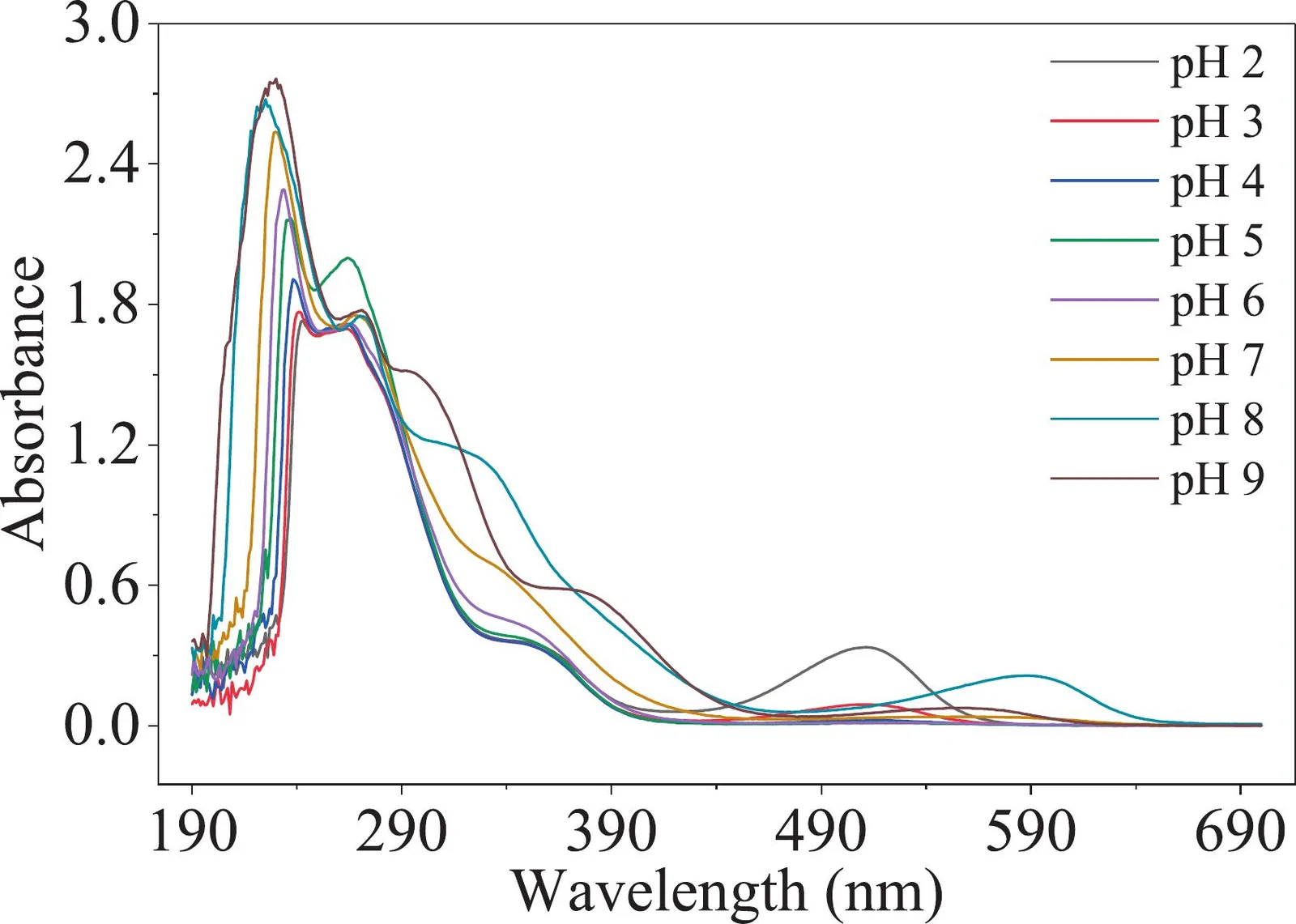
UV–Vis absorption spectra (190–700 nm) showing the characteristic bathochromic shift and hyperchromic effect associated with pH increase.

The effect of pH on the stability of the purified *Rosa chinensis* Jacq. Anthocyanin extract was carried out at varied pH from 2.0 to 9.0. Fig. 5 shows that the extracts were highly susceptible to pH changes as evident from the changes in the color intensity. This spectral behavior is directly illustrated in the UV–Vis absorption spectra (Fig. 6), which show a progressive decrease in absorbance intensity at the visible λ_max_ as pH increased from 2 to 5, consistent with conversion of the colored flavylium cation to the colorless carbinol pseudobase, followed by a bathochromic (red) shift in λ_max_ and partial hyperchromic recovery at pH 6–7, consistent with formation of the violet quinoidal base, and a further bathochromic shift with a broad, diffuse absorption profile at pH 8–9, consistent with progressive structural degradation. Anthocyanins exist in four different chemical forms depending on the pH of the solution, viz., flavylium cation, quinonoidal base, carbinol (pseudobase) and chalcone (Hu et al., 2014; Swer & Chauhan, 2019). At lower pH, anthocyanins primarily exist in the form of flavylium cation contributing to the characteristic red and pink color of the extract (Hu et al., 2014). Increasing the pH deprotonates the flavylium cation forming carbinol (pseudobase) and chalcone that are colorless. These findings are consistent with previously reported pH-dependent structural transitions of cyanidin-based anthocyanins from floral sources (Özgür & Çimen, 2018).

The anthocyanin extracts were found to be more stable at lower pH conditions than higher pH conditions (Table 4). At pH 2, the lowest degradation rate constant and the longest half-life were observed. In contrast, pH 8 exhibited the highest rate constant and the shortest half-life representing a 62-fold increase in degradation rate compared to pH 2. Lower R^2^ values at pH 7–9 reflect both a mechanistic and a kinetic sampling limitation. Mechanistically, alkaline degradation is understood to proceed via multiple concurrent and partially irreversible pathways including chalcone formation, oxidative ring cleavage, and polymerization (Oancea, 2021; Özgür & Çimen, 2018), rather than the single dominant flavylium-to-carbinol equilibrium that governs degradation at acidic pH, making the process inherently less amenable to a simple first-order description. In parallel, the very short half-lives observed at these pH values (4.4–9.4 d) meant that the majority of pigment loss occurred within the first 1–2 d of the 10 d monitoring period, after which retention values plateaued near the assay's detection floor; this compressed the effective dynamic range available for linear regression and further reduced fit quality (Oancea, 2021). At pH 4 to 6, literature confirms that anthocyanins predominantly undergo hydration to form the colorless carbinol pseudobase (Özgür & Çimen, 2018). This drastic reduction in stability at alkaline pH levels is consistent with the behavior of black goji berry anthocyanins, which retain over 90% of their pigment at pH 3 but degrade rapidly as pH increases (Gamage & Choo, 2023). Studies show that under neutral and alkaline conditions, anthocyanins do not follow simple first-order kinetics because they undergo complex, irreversible degradation pathways, including the cleavage of C2-C3 or C3-C4 bonds to form phloroglucinaldehyde and various phenolic acids (e.g., protocatechuic and gallic acids) (Özgür & Çimen, 2018).

#### 3.8.3 Light degradation kinetics

**Fig. 7:**
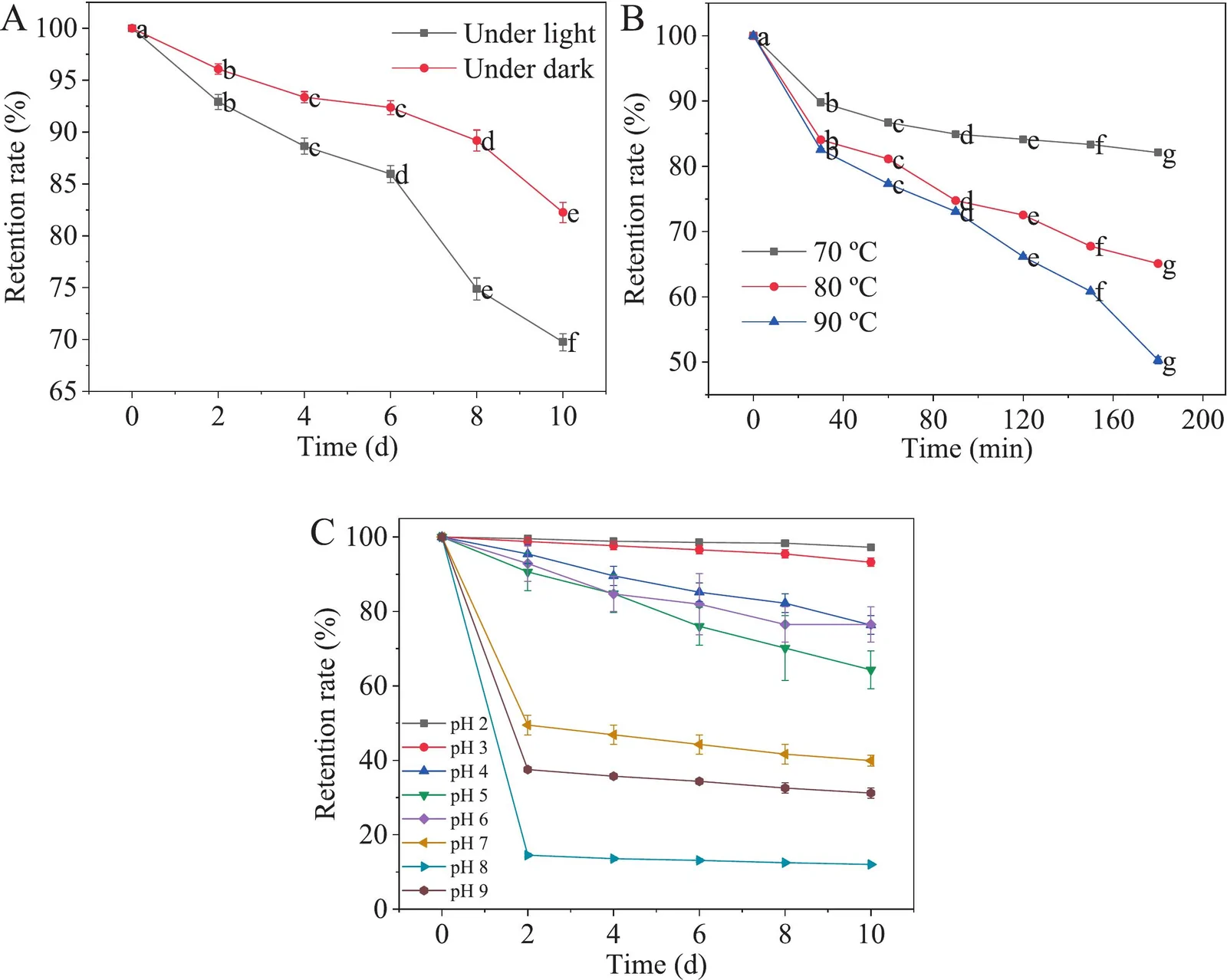
Effect of light (A), temperature (B), and pH (C) on the Pigment retention (%) of purified *Rosa chinensis* Jacq. anthocyanins. Data represent mean ± SD (*n* = 3).

**Fig. 8:**
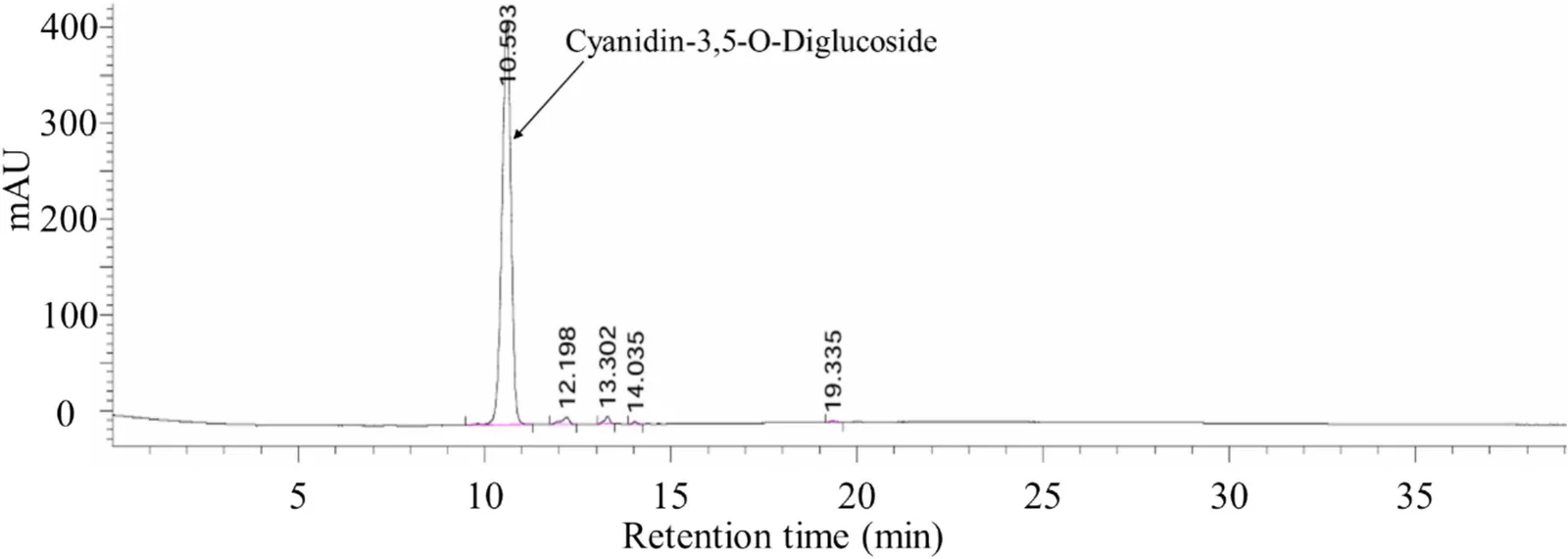
High-performance liquid chromatography (HPLC) chromatogram of the purified anthocyanin extract from *Rosa chinensis Jacq.* petals.

The effect of light exposure was carried out by storing the anthocyanin solution (pH 3.0) under direct light (40 W lamp) and in the dark over ten days. Fig. 7A shows that the extracts were susceptible to light exposure as evident from the differences in pigment retention between the two storage conditions. The anthocyanins stored under light exhibited a continuously decreasing trend in pigment retention, retaining only 69.8% of initial pigment by day 10. In contrast, anthocyanins stored in the dark retained 82.3% of the initial pigment after 10 d. Light accelerates the degradation of anthocyanins primarily through photolytic cleavage of the flavylium cation, generating colorless or brown degradation products such as chalcones and hydroxycinnamic acids (Oancea, 2021; Yücetepe et al., 2024). The energy provided by photons promotes oxidative reactions and ring-opening of the chromophore, which are irreversible processes that permanently diminish color intensity (Li et al., 2023b). These findings are consistent with previously reported light sensitivity of cyanidin-based anthocyanins from floral and fruit sources (Hu et al., 2014; Özgür & Çimen, 2018; Swer & Chauhan, 2019). The greater stability of *Rosa chinensis* anthocyanins under dark storage conditions suggests that opaque or amber-colored packaging would be essential for their practical application as natural colorants in food products. (See Fig. 8.)

Studying the degradation kinetics of anthocyanins with time when subjected to different light conditions indicated a first order reaction (Swer & Chauhan, 2019). The anthocyanin extracts were found to be more stable under dark conditions than under light exposure (Table 4). The outcomes are highly consistent with standardized photostability assays, such as those performed on Lycium ruthenicum (Hu et al., 2014). Anthocyanins extracted from Prunus nepalensis reported half-lives of only 17.3 to 20.3 d in the dark and 9.2 to 9.7 d under light exposure (Swer & Chauhan, 2019). Our extract is approximately twice as stable under both light and dark conditions. Literature confirms that light accelerates deterioration via direct photolytic cleavage and ring-opening of the chromophore, creating a constant, dominant degradation pathway into colorless chalcones, whereas dark storage involves slower, multi-pathway degradation (Yücetepe et al., 2024).

### 3.9 HPLC profiling and purity analysis

The efficacy of resin-based purification was evaluated using HPLC-DAD analysis. The chromatogram recorded at 520 nm (Fig. 7) revealed that the purified *Rosa chinensis* extract consists of a cyanidin-3,5-*O*-diglucoside. The major peak eluted at a retention time (Rt) of 10.59 min, accounting for 97.17% of the total integrated peak area. Based on the elution order characteristic of *Rosa* anthocyanins on a reverse-phase C18 column, where highly polar diglycosides elute earlier than less polar monoglycosides or acylated derivatives, this major compound was identified as Cyanidin-3,5-O-diglucoside (Ge & Ma, 2013). This identification strongly correlates with extensive literature establishing cyanidin-3,5-O-diglucoside as the primary and predominant pigment in red rose petals, frequently reported to account for 90% to over 95% of the total anthocyanin content in red *Rosa chinensis* and *Rosa hybrida* cultivars (Ge & Ma, 2013; Kumari et al., 2022).

Minor peaks were observed at 12.20 min (1.41%) and 13.30 min (1.00%), which likely correspond to pelargonidin derivatives or structural isomers. This is consistent with previous research indicating that pelargonidin derivatives, particularly pelargonidin-3,5-O-diglucoside, are typically the secondary, minor anthocyanins present in red edible roses alongside the dominant cyanidin derivatives (Özgür & Çimen, 2018). The absence of significant noise or broad polymeric humps confirms that the purification process successfully removed the majority of non-anthocyanin impurities and degradation products (Chen et al., 2016).

### 3.10 LC-ESI-MS/MS identification of anthocyanins

**Fig. 9:**
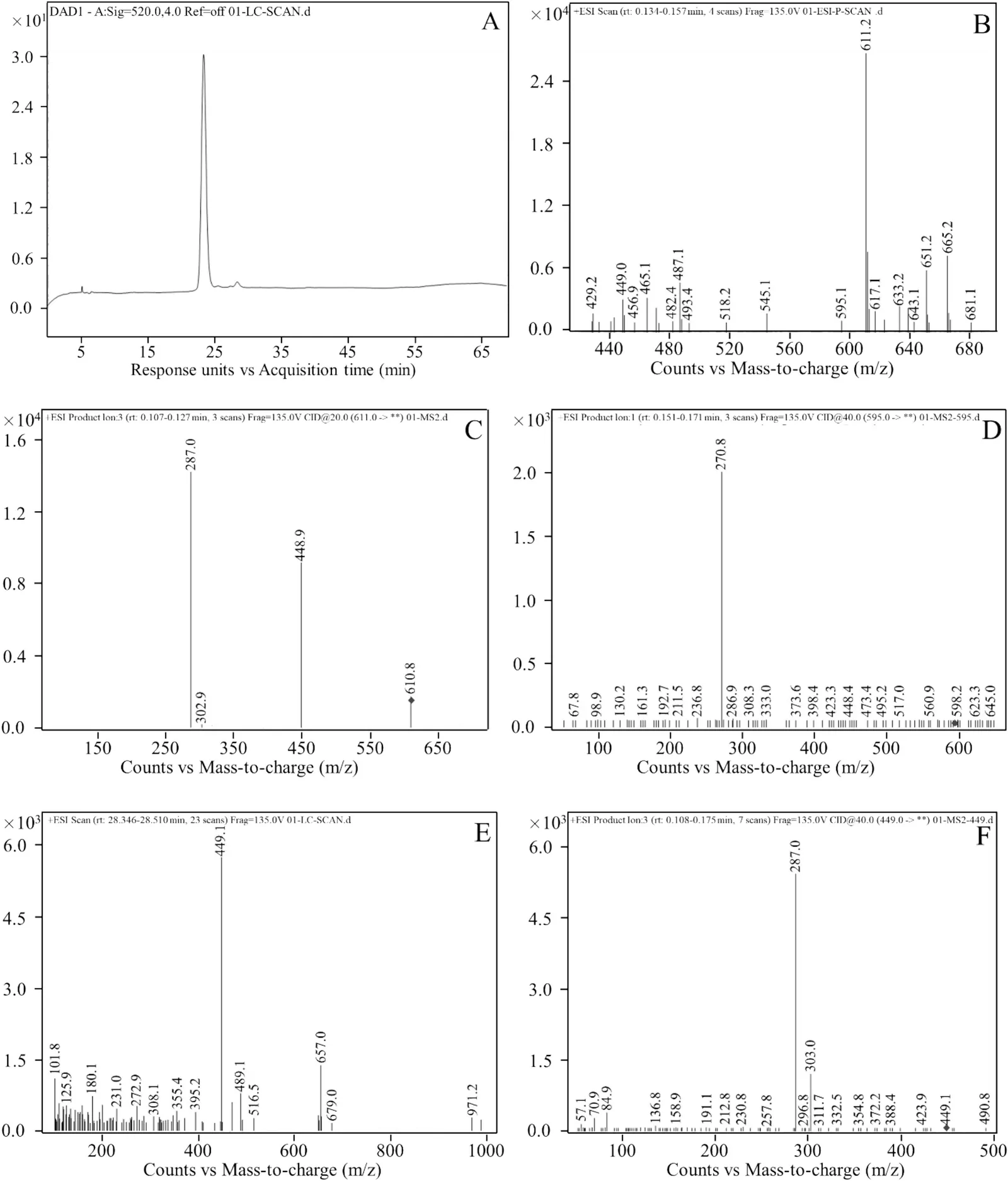
LC-DAD-ESI-MS/MS identification of anthocyanins in purified *Rosa chinensis* flower extract. (A) UV chromatogram at 520 nm showing three anthocyanin peaks. (B) Full-scan ESI-MS showing dominant [M + H]^+^ at *m*/*z* 611.2 (cyanidin-3,5-O-diglucoside) with minor ions at *m*/*z* 595.1 and 449.0. (C) MS/MS of *m*/*z* 611.0 at 20 eV showing fragments at *m*/*z* 448.9 (−162 Da) and 287.0 (cyanidin aglycone, −324 Da), confirming the 3,5-diglucoside structure. (D) MS/MS of *m*/*z* 595.0 at 40 eV showing *m*/*z* 270.8 (pelargonidin aglycone), confirming pelargonidin-3,5-O-diglucoside. (E) LC full-scan at RT 28.3–28.5 min showing *m*/*z* 449.1 (cyanidin-3-O-glucoside). (F) MS/MS of *m*/*z* 449.0 at 40 eV showing *m*/*z* 287.0 (cyanidin aglycone, −162 Da), confirming the monoside structure. All identifications at MSI Level 2.

Structural identification of the three main anthocyanins were achieved by the LC-ESI-MS/MS in positive ion mode, with in-source fragmentation voltage set at 135 V. The fragmentation data obtained at multiple collision energies (20, 30, and 40 eV) are presented in Fig. 9.

The full-scan positive ESI mass spectrum from the LC-MS acquisition revealed a dominant molecular ion [M + H]^+^ at *m*/*z* 611.2, which is consistent with the molecular formula C₂₇H₃₁O₁₆^+^ (calculated 611.16) corresponding to cyanidin-3,5-O-diglucoside (Fig. 9B). This was confirmed by MS/MS fragmentation at 20 eV (Fig. 9C), which produced two diagnostic fragment ions: *m*/*z* 449.0 (corresponding to [M + H − 162.05]^+^, loss of 5-O-glucose) and *m*/*z* 287.0 (corresponding to the cyanidin aglycone, C₁₅H₁₁O₆^+^, calculated 287.055, after a second neutral loss of 3-O-glucose, −162.05 Da). The simultaneous appearance of both intermediate (*m*/*z* 449.1) and terminal (*m*/*z* 287.0) fragment ions at 20 eV demonstrated the sequential two-step neutral loss pathway: 611.2 → 449.0 (−162.05 Da) → 287.0 (−162.05 Da), which is the characteristic fragmentation cascade diagnostic of the 3,5-O-diglucoside configuration (Barnes & Schug, 2011). At higher collision energy (40 eV), the cyanidin aglycone at *m*/*z* 287.0 predominated as the base peak, confirming complete sequential glycan loss. This identification is consistent with previously published LC-MS/MS data for cyanidin-3,5-O-diglucoside in red and pink Rosa cultivars (Mikanagi et al., 2000; Wan et al., 2019) and confirms and upgrades the tentative Level 3 assignment previously reported for this species by wide-target metabolomics profiling.

The second anthocyanin peak (retention time ∼ 25 min, 1.41% of 520 nm area) showed a molecular ion [M + H]^+^ at *m*/*z* 595.1 in the full-scan spectrum (Fig. 9B), consistent with pelargonidin-3,5-O-diglucoside (C₂₇H₃₁O₁₅^+^, calculated 595.17). MS/MS at 40 eV (Fig. 9D) yielded an unambiguous base fragment at *m*/*z* 270.8 ≈ 271.0, corresponding to the pelargonidin aglycone (C₁₅H₁₁O₄^+^, calculated 271.06) after sequential neutral loss of two glucose units (2 × 162.05 Da = 324.10 Da). The absence of *m*/*z* 287.0 in this spectrum and the exclusive presence of *m*/*z* 271.0 unambiguously differentiated this compound from the cyanidin-based dominant peak, confirming the pelargonidin B-ring (monohydroxy, 4’-OH) versus the cyanidin catechol B-ring (3′,4’-diOH). MS/MS of the 595.0 precursor at 20 eV also showed 271.0 as the dominant fragment, indicating that the two-step glycan loss is highly efficient for this compound even at low collision energy, consistent with the lability of the glycosidic bonds in pelargonidin derivatives (Wan et al., 2019).

The third anthocyanin (retention time ∼ 28 min) was identified in the LC full-scan spectrum at this retention time window (Fig. 9E) as a base peak at *m*/*z* 449.1, consistent with cyanidin-3-O-glucoside (C₂₁H₂₁O₁₁^+^, calculated 449.108). MS/MS at 40 eV (Fig. 9F) yielded a dominant fragment at *m*/*z* 287.0 (cyanidin aglycone) after a single neutral loss of 162.05 Da (one glucose unit), confirming the 3-O-monoside structure. The single-step fragmentation (449.1 → 287.0, −162.05 Da) clearly distinguished this compound from cyanidin-3,5-O-diglucoside and confirmed its assignment as cyanidin-3-O-glucoside. The later elution of this compound (RT ∼28 min) compared with the 3,5-O-diglucosides (∼23 and ∼ 25 min) is consistent with its lower polarity on the C18 reversed-phase column: the absence of the 5-O-glucose reduces the overall hydrophilicity, resulting in stronger hydrophobic interaction with the stationary phase and therefore longer retention. This elution order is consistent with established anthocyanin chromatographic behavior on reversed-phase C18 columns under acidic mobile phase conditions (Avula et al., 2023).

The three anthocyanins identified were assigned at MSI confidence Level 2 (Sumner et al., 2007) based on accurate molecular ion masses and diagnostic MS/MS fragmentation patterns consistent with published reference data, in the absence of authentic reference standard co-injection. The identification of cyanidin-3,5-O-diglucoside as the dominant anthocyanin (97.17% of 520 nm area) is in full agreement with established reports for red and pink Rosa cultivars, in which cyanidin-3,5-O-diglucoside is characteristically the principal pigment (Kumari et al., 2022; Mikanagi et al., 2000). The co-occurrence of pelargonidin-3,5-O-diglucoside and cyanidin-3-O-glucoside as minor anthocyanins is also consistent with previously reported profiles for hybrid tea rose varieties, where multiple aglycone types may coexist at low concentrations alongside the dominant cyanidin diglucoside (Wan et al., 2019).

## 4 Conclusions

This study demonstrates that ultrasound-assisted extraction, optimized via a Box-Behnken response surface design, is an efficient, green strategy for recovering anthocyanins from *Rosa chinensis* Jacq. petals, with extraction temperature and time identified as the dominant process variables. Using a food-grade acidified ethanol solvent system, the optimized protocol achieved a high yield (34.99 g kg^−1^ DW) without reliance on toxic solvents. Purification and LC-ESI-MS/MS characterization confirmed cyanidin-3,5-O-diglucoside as the principal pigment, identified at MSI confidence Level 2, alongside minor pelargonidin- and cyanidin-glucoside derivatives. The purified extract combined strong antioxidant activity with favorable stability under pasteurization-relevant thermal conditions, acidic pH, and dark storage. Stability was strongly pH- and light-dependent, with degradation rate increasing ∼62-fold from pH 2 to pH 8, and dark storage preserving substantially more pigment than light exposure over ten days (82.3% vs. 69.8%). Collectively, these findings support the potential of *Rosa chinensis* petals as a sustainable, functional source of natural anthocyanin colorants and antioxidants for food and nutraceutical applications, particularly in acidic, heat-treated, or light-protected product formats such as beverages, dairy products, and confectionery.

This study was limited to a single rose cultivar and to laboratory-scale extraction and stability assessment under model solvent systems rather than complex food matrices; anthocyanin behavior in real food systems can be influenced by matrix interactions (e.g., proteins, sugars, metal ions) not captured here. Future work should evaluate the performance and stability of these anthocyanins upon direct incorporation into target food products, assess scale-up feasibility of the extraction and purification process, and examine encapsulation strategies to further improve light and pH stability for commercial application.

## Declaration of generative AI and AI-assisted technologies in the manuscript preparation process

During the preparation of this manuscript, the author(s) utilized ChatGPT to assist with language refinement, including grammar, style, and clarity. All content was carefully reviewed, edited, and verified by the author(s), who accept full responsibility for the accuracy, integrity, and originality of the final published work.

## CRediT authorship contribution statement

**Naeem Arshad:** Writing – review & editing, Writing – original draft, Methodology, Investigation, Data curation. **Aroona Maryam:** Methodology, Investigation, Data curation. **Zetian Cai:** Methodology, Investigation, Data curation. **Haowen Meng:** Methodology, Investigation, Data curation. **Bingxue Wang:** Methodology, Investigation, Data curation. **Xiaoxue Kong:** Methodology, Data curation. **Haibo Luo:** Writing – review & editing, Supervision, Project administration, Conceptualization. **Lijuan Yu:** Writing – review & editing, Funding acquisition, Conceptualization, Supervision.

## Declaration of competing interest

The authors declare that they have no known competing financial interests or personal relationships that could have appeared to influence the work reported in this paper.

## Data Availability

Data will be made available on request.
